# The effect of varying analytical methods on estimates of anti-malarial clinical efficacy

**DOI:** 10.1186/1475-2875-8-77

**Published:** 2009-04-22

**Authors:** Wendy J Verret, Grant Dorsey, Francois Nosten, Ric N Price

**Affiliations:** 1 Department of Epidemiology, School of Public Health, University of California, Berkeley, California, USA; 2 Department of Medicine, San Francisco General Hospital, University of California, San Francisco, California, USA; 3 Shoklo Malaria Research Unit, Mae Sot, Thailand; 4 Faculty of Tropical Medicine, Mahidol University, Bangkok, Thailand; 5 Centre for Clinical Vaccinology and Tropical Medicine, Nuffield Department of Clinical Medicine, Churchill Hospital, Oxford, UK; 6 International Health Program, Menzies School of Health Research and Charles Darwin University, Darwin, Northern Territory, Australia

## Abstract

**Background:**

Analytical approaches for the interpretation of anti-malarial clinical trials vary considerably. The aim of this study was to quantify the magnitude of the differences between efficacy estimates derived from these approaches and identify the factors underlying these differences.

**Methods:**

Data from studies conducted in Africa and Thailand were compiled and the risk estimates of treatment failure, adjusted and unadjusted by genotyping, were derived by three methods (intention to treat (ITT), modified intention to treat (mITT) and per protocol (PP)) and then compared.

**Results:**

29 clinical trials (15 from Africa and 14 from Thailand) with a total of 65 treatment arms (38 from Africa and 27 from Thailand) were included in the analysis. Of the 15,409 patients enrolled, 2,637 (17.1%) had incomplete follow up for the unadjusted analysis and 4,489 (33.4%) for the adjusted analysis. Estimates of treatment failure were consistently higher when derived from the ITT or PP analyses compared to the mITT approach. In the unadjusted analyses the median difference between the ITT and mITT estimates was greater in Thai studies (11.4% [range 2.1–31.8]) compared to African Studies (1.8% [range 0–11.7]). In the adjusted analyses the median difference between PP and mITT estimates was 1.7%, but ranged from 0 to 30.9%. The discrepancy between estimates was correlated significantly with the proportion of patients with incomplete follow-up; p < 0.0001. The proportion of studies with a major difference (> 5%) between adjusted PP and mITT was 28% (16/57), with the risk difference greater in African (37% 14/38) compared to Thai studies (11% 2/19). In the African studies, a major difference in the adjusted estimates was significantly more likely in studies in high transmission sites (62% 8/13) compared to studies in moderate transmission sites (24% 6/25); p = 0.035.

**Conclusion:**

Estimates of anti-malarial clinical efficacy vary significantly depending on the analytical methodology from which they are derived. In order to monitor temporal and spatial trends in anti-malarial efficacy, standardized analytical tools need to be applied in a transparent and systematic manner.

## Background

In the past decade, the number of anti-malarial clinical trials has increased significantly. In Africa alone, the number of such studies published between 2001 and 2005 increased three-fold compared to the number published in the preceding five years [1]. This increase is primarily due to greater awareness of the emergence of multidrug resistant strains of *Plasmodium falciparum *and to the introduction of new treatment regimens such as artemisinin combination therapy (ACT). In addition, study designs have evolved to include a longer duration of follow-up and the inclusion of genotyping to distinguish recrudescence from new infection [1].

Anti-malarial clinical trials are usually conducted either to compare two or more treatment regimens (comparative trials) or to monitor for the emergence of anti-malarial resistance over time and in different geographical areas. The World Health Organization (WHO) currently recommends that countries change their anti-malarial treatment policy when the cure rate for the current recommended therapy falls below 90% and that a new anti-malarial treatment policy be adopted only when a therapy has an average cure rate ≥ 95% [2]. The WHO also recommends the use of survival analysis to generate efficacy estimates; however, in practice researchers adopt a variety of statistical methods tailored to the rationale of the specific study [3-5]. The derived estimates are confounded further by variations in the PCR correction methods used to distinguish recrudescent infections from new infections [1,6]. These methodological differences undermine attempts to monitor and compare cure rates between locations and over time and significantly limit the utility of clinical trials to guide policy [7].

In general, anti-malarial efficacy can be calculated using three approaches: per protocol, intention-to-treat and modified intention-to-treat. In the per protocol analysis (PP) the evaluable population includes only those patients who are followed throughout the protocol-defined follow-up period and in whom a clear treatment outcome can be determined. In this approach, patients deviating from the protocol, such as those who do not complete follow-up, are excluded from the analysis. Intention-to-treat analysis (ITT) adopts a conservative approach often advocated for comparative drug trials, in which all patients randomized to treatment are included in the analysis and patients with incomplete follow-up who do not reach the primary outcome of interest are generally considered treatment failures. In the third approach, the modified intention-to-treat analysis (mITT), survival analysis is used and patients with incomplete follow-up who do not reach the primary outcome of interest are included in the analysis as non-failures, but censored on the last day of follow-up. WHO guidelines and several recent consensus papers advocate modified ITT survival analysis as the most appropriate method for monitoring anti-malarial efficacy [3,5,7,8].

The aim of the current study was to quantify the magnitude of the differences between efficacy estimates derived from survival analysis of a mITT approach with that of simple proportions from PP and ITT approaches and to identify factors that influence these differences. Data were compiled from 29 comparative anti-malarial clinical trials conducted in Africa and Thailand and the derived estimates of treatment failure compared.

## Methods

### Data sources for analysis comparisons

Individual patient data were available from 14 comparative clinical trials conducted in Thailand between 1993 and 2005 and from 15 comparative clinical trials conducted in Uganda and Burkina Faso between 2003 and 2007 (Additional File 1). Data were included only for patients enrolled with uncomplicated malaria due to *P. falciparum*. Drug treatment was supervised in all patients, with daily observation until at least day 3 followed by weekly visits up to 28, 42, or 63 days.

### Thai studies

The studies in Thailand were carried out in a camp for displaced persons of the Karen ethnic minority on the western border of Thailand [9]. Transmission of malaria here is unstable and seasonal, with peaks in May through July and December through January [10]. The estimated entomological inoculation rate (EIR) and corresponding incidence of malaria is low (approximately 0.5 to 1.5 cases/person/year), with prevalence rates of 1–4% for *P. falciparum*. Overall, *P. falciparum *accounts for 37% of malaria infections, with the remainder due to *P. vivax*. All *P. falciparum *infections and approximately 90% of *P. vivax *infections are symptomatic. In Thailand, patients of all ages were enrolled, providing that they weighed more than five kilograms. Pregnant women and patients with severe disease were excluded.

### African studies

The studies in Africa were conducted in Bobo-Dioulasso, Burkina Faso and in several study sites in Uganda. Patients recruited were six months of age or older with no evidence of severe disease. *Plasmodium falciparum *accounts for nearly 100% of all malaria cases in these regions. In Burkina Faso, malaria is seasonal, with transmission peaking during the rainy season from May to October. All patients were recruited from governmental health clinics. Studies in Uganda were conducted in areas of moderate to high transmission intensity, with peaks during two rainy seasons from March to May and from August to September. Three studies, in Kampala, Apac, and Tororo, were conducted in children only. Patients were recruited from district health clinics participating in the Ugandan Malaria Surveillance Project, household sampling, or from other outpatient clinics.

### Malaria outcome classification

The key parameters for deriving the efficacy estimates were coded identically for all studies, as described previously [7]. Outcomes were classified according to the 2006 WHO guidelines as adequate clinical and parasitological response (ACPR), early treatment failure (ETF), late clinical failure (LCF), late parasitological failure (LPF), or follow-up interrupted (Table 1). For 24 of the 29 (83%) studies, parasites were genotyped to distinguish recrudescent and new infections due to *P. falciparum*, as previously described [1,11]. All ETFs were considered to be due to recrudescence. Patients meeting the criteria for LCF or LPF in whom genotyping was done but results were inconclusive or unavailable were classified as unsuccessfully genotyped.

**Table 1:** Treatment outcome classification system using standardised criteria [7].

**Outcome Category**	**Outcome Code**	**Outcome Definition**	**Africa**	**Thailand**	**Total**
**Follow-up completed**	0	ACPR	4385	4604	8989
	
	1	ETF with death	0	1	1
	
	2	ETF with severe malaria	5	0	5
	
	3	ETF with danger signs	39	0	39
	
	4	ETF with parasitological criteria	58	1	59
	
	5	ETF with clinical criteria	9	0	9
	
	6	ETF not otherwise specified	0	11	11
	
	7	LCF with death	0	0	0
	
	8	LCF with severe malaria	0	0	0
	
	9	LCF with danger signs	4	0	4
	
	10	LCF with fever	1033	654	1687
	
	11	LPF	1726	665	2391
	
	12	LPF/LCF indistinguishable	0	737	737

**Follow-up interrupted**	13	Adverse event requiring change in anti-malarial therapy	0	2	2
	
	14	Treatment protocol violation	4	138	142
	
	15	Death not due to malaria	0	3	3
	
	16	Lost to follow-up	175	955	1130
	
	17	Use of other anti-malarials outside of study protocol	48	6	54
	
	18	Withdrawal of consent prohibiting further follow-up	126	1	127
	
	19	Investigator initiated withdrawal from further follow-up	7	0	7
	
	20	Patient who does not complete follow-up for any other reason	0	12	12

ACPR = adequate clinical and parasitological response; ETF = early treatment failure; LCF = late clinical failure; LPF = late parasitological failure

### Statistical analyses

The risks of failure unadjusted and adjusted by genotyping for each treatment arm of the individual studies were derived and compared using three analytical methods; per protocol (PP), intention-to-treat (ITT), and modified intention-to-treat (mITT). Although the general principles behind these analytical approaches are well-described, in practice subtle differences arise in the way in which the outcome measures may be classified. For the purpose of the present analysis, treatment outcomes were classified as summarized in Table 2. In the ITT analyses, the evaluable population for both the unadjusted and adjusted calculations included all patients enrolled in the study. In the PP analysis, the evaluable population included only patients classified as ACPR or recurrent parasitemia with *P. falciparum *(ETF, LPF, LCF) in the unadjusted calculations and only patients classified as ACPR, ETF or LCF/LPF due to recrudescence in the adjusted calculations. In the mITT analyses, the evaluable population for both the unadjusted and adjusted calculations included all patients enrolled in the study, with the exception that LCF/LPF outcomes with unsuccessful genotyping outcomes were excluded from the adjusted calculations. In the PP and ITT analyses, the risk of failure for each treatment group was calculated as the proportion of patient classified as failure (the numerator) divided by the number of patients in the evaluable population (the denominator). In the mITT analyses, the risk of failure was calculated using the Kaplan-Meier product limit formula with data censored for patients who were not classified as failures and with interrupted follow-up. For the unadjusted calculations, patients with follow-up interrupted and non-falciparum new infections were censored on the last day of observation. For the adjusted calculations, censored patients also included those with new *P. falciparum *infections. Risk of failure estimates derived from the three analytical methods are provided in Additional File 2.

**Table 2:** Analytical methods used to generate estimates of anti-malarial drug efficacy.

	**Outcome Category**	**Unadjusted by genotyping**			**Adjusted by genotyping**		
		**ITT**	**mITT**	**PP**	**ITT**	**mITT**	**PP**
	**Follow-up interrupted**	Failure	Censored	Excluded	Failure	Censored	Excluded
	**ACPR**	Success	Success	Success	Success	Success	Success
	**ETF**	Failure	Failure	Failure	Failure	Failure	Failure
**LCF and LPF**	**Recrudescence**	Failure	Failure	Failure	Failure	Failure	Failure
	**P. falciparum new infection**	Failure	Failure	Failure	Success	Censored	Excluded
	**Genotyping unsuccessful**	Failure	Failure	Failure	Failure	Excluded	Excluded
	**Non-falciparum new infection**	Success	Censored	Excluded	Success	Censored	Excluded

ITT = intention-to-treat; mITT = modified intention-to-treat; PP = per protocol

ACPR = adequate clinical and parasitological response; ETF = early treatment failure; LCF = late clinical failure; LPF = late parasitological failure

Note: Although the general principles behind these analytical approaches are well-described, in practice differences arise in the way in which the outcome measures may be classified, depending on the rationale of the study. The Table reflects only one set of options and is not definitive for all antimalarial studies.

The relationship between the proportion of patients with incomplete follow-up and the risk difference were compared using two different methods for estimating the risk of failure. Incomplete follow-up included any outcome category (listed in Table 2), where the classification of success/failure/censored/excluded differed between any of the three analytical methods. In the unadjusted analyses, incomplete follow-up was defined as any patient in whom follow-up was interrupted and those with non-falciparum new infections. In the adjusted analyses, incomplete follow-up was defined as any patient in whom follow-up was interrupted, those with non-falciparum new infections, those with *P. falciparum *new infections, and those with unsuccessful genotyping.

Since there were exclusive differences in the study characteristics between Thailand and Africa, stratified analyses were used to evaluate factors that may contribute to the pairwise differences in the risk of failure between the analytical methods for both the adjusted and unadjusted calculations. The following potential factors associated with incomplete follow up were included in the analysis: the location of the study (Africa or Thailand), the duration of study follow-up (28, 42, or 63 days), and malaria transmission intensity (classified as low (EIR < 1), moderate (EIR 1 to 100) and high (EIR > 100)).

All analyses were performed with Stata, version 10 (Stata-Corp, College Station, Texas). A p-value < 0.05 was considered statistically significant.

## Results

In total, 29 drug studies were included in the analysis, with 65 treatment arms that enrolled 15,409 patients. Five (17%) trials in Thailand that included eight treatment arms were conducted prior to the introduction of genotyping and thus were not included in the adjusted analyses. Of the 15 studies conducted in Africa, the duration of follow-up was 28 days in 12 (80%) studies and 42 days in 3 (20%) studies. Ten (66%) trials conducted in Africa were conducted in areas of moderate transmission intensity and the remainder were conducted in areas of high transmission. Of the 14 studies conducted in Thailand, the duration of follow-up was 28 days for one (7%) study, 42 days for five (36%) studies, and 63 days for eight (57%) studies. All Thai studies were conducted in an area of low intensity transmission. Clinical outcomes for each location (Africa and Thailand) are summarized in Table 1.

### Incomplete follow-up

For analyses unadjusted by genotyping, incomplete follow can be divided into two categories: patients whose follow-up is interrupted prior to reaching a defined endpoint (i.e. lost to follow-up) and recurrent malaria due to non-falciparum infections (Table 3). In total, 29% (2,237/7,790) of Thai patients had incomplete follow-up for the unadjusted risk estimates, of which 50% (1,120/2,237) had follow-up terminated early due to recurrence with *P. vivax*. Incomplete follow-up was significantly less frequent in African studies (5.3% 400/7,619, p < 0.001), with only 10% (40/400) of patients with incomplete follow-up having recurrence with a different species. For the adjusted analyses, patients with new *P. falciparum *infections and recurrent infections that could not be genotyped were also classified as having incomplete follow-up. These outcomes occurred in 26% (1971/7619) of African patients, but only 7.3% (422/5813) of Thai patients; p < 0.0001.

**Table 3:** Proportion of patients with incomplete follow-up.

	**Africa**	**Thailand**	**Overall**
**Unadjusted Analysis**	7619	7790	15,409
**Interrupted follow-up**^a^	360 (4.7%)	1117 (14.3%)	1477 (9.6%)
**Non-falciparum new infections**	40 (0.5%)	1120 (14.4%)	1160 (7.5%)
**Overall**	**400 (5.3%)**	**2237 (28.7%)**	**2637 (17.1%)**
**Adjusted Analysis**^b^	7619	5813	13,432
**Interrupted follow-up**^a^	360 (4.7%)	673 (11.6%)	1033 (7.7%)
**Non-falciparum new infections**	40 (0.5%)	1023 (17.6%)	1063 (7.9%)
***P. falciparum *new infections**	1864 (24.5%)	323 (5.6%)	2187 (16.3%)
**Unsuccessful genotyping**	107 (1.4%)	99 (1.7%)	206 (1.5%)
**Overall**	**2371 (31.1%)**	**2118 (36.4%)**	**4489 (33.4%)**

^a ^Includes all patients with interrupted follow-up (outcomes 13–20 in table 1)

^b ^Excludes 1977 Thai patients (from 5 studies with 8 treatment arms) in which genotyping was not attempted

In Thailand, studies with a longer duration (42 days or more) had a greater proportion of patients with incomplete follow-up compared to studies with only 28-days of follow-up. This was apparent for both the unadjusted (median 22% vs 27%; p = 0.028) and adjusted analyses (median 22% vs 35%; p = 0.004). In Africa, where there was variation in transmission intensity, incomplete follow-up in the adjusted analyses was significantly higher in areas of high transmission (median 47% [range: 32.2–68.6]) compared to studies in moderate transmission areas (19% [9.9–48.2]; p < 0.001). Patients with new *P. falciparum *infections accounted for 91% (1101/1204) of the patients with incomplete follow-up in high transmission areas, compared to 65% (763/1,167) in moderate transmission areas; p < 0.0001. Conversely, in the unadjusted analyses, the proportion of patients with incomplete follow-up was low in both high transmission sites (median 1.9% [range 0.6–6.2]) and moderate transmission sites (median 4.5% [range 2.2–12.8]).

### Comparison of ITT and mITT analyses

**Figure 1:**
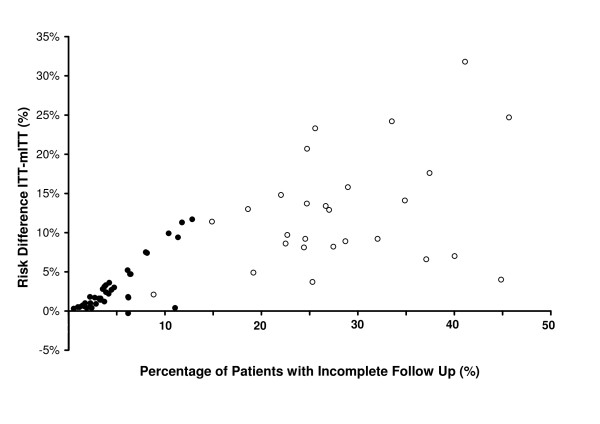
**Relationship between incomplete follow up and the risk difference between unadjusted estimates from ITT and mITT analysis**. Closed circles for African studies (38 treatment arms) and open circles for Thai studies (27 treatment arms).

The unadjusted risk of treatment failure derived by ITT analysis was consistently higher than that derived by mITT analysis (median difference = 4.7% [-0.3–31.8%]) (Table 4). The difference in risk estimates (ITT-mITT) was greater in Thai studies (median = 11.4 [range 2.1–31.8]) compared to African Studies (median 1.8% [range 0–11.7]; p < 0.001). The difference between the unadjusted risk estimates was correlated with the proportion of patients with incomplete follow-up in African studies (r_s _= 0.721, p < 0.0001), although this does not reach significance in the Thai studies (r_s _= 0.272, p = 0.169); Figure 1. The ITT-mITT risk difference was higher for the adjusted estimates compared to the unadjusted estimates, and this was apparent for both the African studies (median 3.5% vs 1.8%) and the Thai studies (median 12.3% vs. 11.4%). In Africa, 18% (7/38) of treatment arms had a difference in the unadjusted risk estimates (ITT-mITT) greater than 5%, compared to 85% (23/27) of the studies in Thailand; p < 0.001. The corresponding figures for the difference in the adjusted estimates were 29% (11/38) and 95% (18/19) respectively, p < 0.001.

**Table 4:** The difference in risk estimates derived by intention to treat (ITT), modified Intention to Treat (mITT), and per protocol (PP).

	**Africa**	**Thailand**	**Overall**
**ITT – mITT**			
**Unadjusted**	1.8% [-0.3–11.7] IQR: 0.7–3.9	11.4% [2.1–31.8]^a^ IQR: 8.1–15.8	4.7% [-0.3–31.8] IQR: 1.6–10.6
**Adjusted**	3.5% [-13.7–14.4] IQR: 0.9–6.0	12.3% [4.1–31.8]^a^ IQR: 10.2–16.0	5.4% [-13.7–31.8] IQR: 1.9–11.6
**PP – mITT**			
**Unadjusted**	0.1% [0.0–2.1] IQR: 0–0.23	1.9% [0.0–10.6]^a^ IQR: 0.9–4.3	0.3% [0.0–10.6] IQR: 0.1–1.9
**Adjusted**	3.2% [0.0–0.9] IQR: 0.7–3.9	1.0% [0.0–6.9]^b^ IQR: 0.3–1.8	1.7% [0.0–30.9] IQR: 0.5–5.6

Values represent Median [Range], and InterQuartile Range (IQR)

Comparison between Africa and Thailand: ^a ^p < 0.001; ^b ^p = 0.033

### Comparison of PP and mITT analyses

The unadjusted risk of treatment failure derived from the PP analyses was consistently higher than that derived from the mITT analyses (Table 4). The median difference (PP-mITT) in Thailand was 1.9% (range 0–10.6) and was correlated with both the proportion of patients with incomplete follow-up (p = 0.02) and the duration of the study (p = 0.03). The difference in the estimates was significantly smaller in African studies (median = 0.1% [range 0 to 2.1%]; p < 0.001), and was correlated with the study duration (p = 0.005).

**Figure 2:**
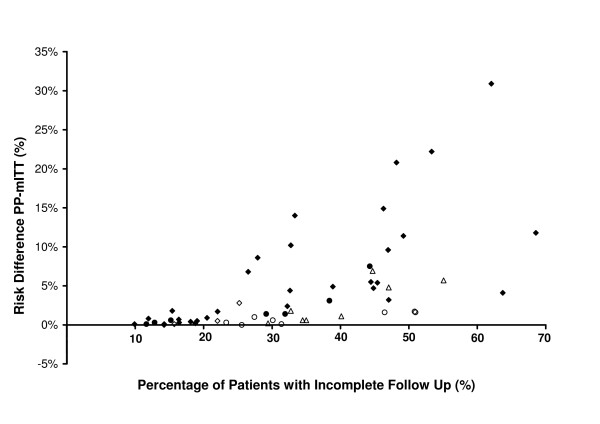
**Relationship between incomplete follow up and the risk difference between adjusted estimates from PP and mITT analysis**. Closed markers for African studies (38 treatment arms) and open for Thai studies (19 treatment arms). Diamonds = 28 day studies, Circles = 42 days studies and triangles 63 days studies.

In the adjusted analyses the median difference between estimates was 1.7% (range 0–30.9) and was correlated significantly with the proportion of patients with incomplete follow-up (p < 0.0001; Figure 2) in both Africa and Thailand. The difference was greater in Africa (median 3.2% [range 0–30.9]) compared to Thailand (median 1.0% [range 0–6.9]; p = 0.033).

In total, 7.7% (5/65) of the treatment arms had a difference in the unadjusted risk estimates (PP-mITT) of greater than 5%; these studies were all from Thailand (19% 5/27), with none (0/38) conducted in Africa; p = 0.01. In the adjusted analyses the proportion with a major difference (> 5%), rose to 28% (16/57), with the risk greater in African (37% 14/38) compared to Thai studies (11% 2/19). In Africa the likelihood of a major difference in adjusted estimates was significantly greater in studies conducted in high transmission sites (62% 8/13) compared to moderate transmission sites (24% 6/25); p = 0.035.

## Discussion

Anti-malarial drug clinical trials are conducted both to monitor anti-malarial drug resistance and to compare treatment regimens. As in all clinical trials, protocol violations and incomplete patient follow-up challenge the analysis and interpretation of the results. Malaria studies are, by their nature, logistically difficult, often being conducted in poorly resourced communities and prone to varying patient adherence to protocols. In addition to problems related to protocol adherence, anti-malarial clinical trials are also confounded by interrupted follow-up resulting from recurrent infections, either by the same or different malaria species. The statistical approach to deal with these challenges can vary according to the rationale of the study [3,12]. For instance, in comparative studies a conservative approach (i.e. intention to treat, ITT), in which all patients are included in the analysis but those with incomplete follow-up are classified as a treatment failure, is often advocated. In contrast, when monitoring anti-malarial drug resistance, the objective is to determine the risk of failure, with failure limited to those with a clearly inadequate response to therapy. Patients with incomplete follow-up can be either dropped from the analysis (e.g. per protocol, PP), or included in a survival analysis with censoring as "non-failures" on the last day of follow-up (modified intention to treat, mITT). The WHO currently recommends the latter as the preferred method of analysis of malaria drug efficacy studies [8], although accepts the option of per protocol analysis. In this paper, three analytical methods were compared from drug trials conducted in Thailand, Uganda, and Burkina Faso to determine the degree of variation in the derived estimates of efficacy and factors underlying this.

The studies presented come from two highly experienced research groups, and although the proportion of patients with interrupted follow-up (i.e. incomplete follow-up due to reasons other than recurrent infections) was generally low, this rose to as high as 36%. Interrupted follow-up was greater in the Thai studies compared to those conducted in Africa, in part because of the longer duration of study follow-up in Thailand. The occurrence of new *P. falciparum *infections or relapse of *P. vivax *infection, generally require retreatment and termination of the primary study. Even in study populations with the highest adherence rates, these proportions can often exceed a third of all patients enrolled (Additional File 1), reducing considerably the per protocol population. Predictably incomplete follow-up was higher for the adjusted estimates, which distinguishes recurrent infections, and in the African studies this was more apparent in studies conducted in areas of high transmission.

The proportion of patients with incomplete follow-up has significant implications for the derived estimates of treatment efficacy. Both the ITT and PP methods consistently over-estimated the risk of failure when compared to the preferred mITT method, the discrepancy in risk estimates varying from trivial to highly significant. For example, in the comparison of the unadjusted ITT and mITT failure estimates, 46% (30/65) of the difference in estimates exceeded 5%, with one study having a difference of 31.8%. The bias was most pronounced in Thailand due to the high percentage of patients with incomplete follow-up. These findings highlight that although the ITT method of analysis has utility for conservatively comparing treatment arms within a comparative drug trial, it is significantly biased when deriving point estimates of efficacy, for comparison over time or geographical location.

New infection with *P. falciparum *constituted an additional confounding factor for the adjusted analyses (PP-mITT). Whereas individuals with such infections are removed from the PP analysis, they are censored in the mITT analysis after contributing a period of observation to the cumulative risk during which treatment failure was not observed. As a consequence, the PP analysis consistently overestimates treatment failure compared to that derived by the mITT survival analysis (median = 1.7% [IQR 0.5–5.6]). In 28% (16/57) of cases this difference exceeded an absolute value of 5%. The discrepancy was particularly apparent in the high transmission sites in Africa where reinfections were highest. The differences in risk estimates were lower for the unadjusted analyses, although in Thailand, high relapse rates with *P. vivax *and greater loss to follow-up resulted in 18.5% (5/27) of PP estimates deviating by more than 5% from the mITT estimate.

Survival analysis is being used increasingly to derive estimates of anti-malarial treatment efficacy. Although the ease of calculating the simple proportions in the PP analysis retains its appeal, and these estimates continue to be reported frequently in the literature, caution is needed when generating temporal and geographical trends using different analytical methods. This is particularly true for studies with poorer patient adherence to follow-up, higher incidence of *P. vivax *relapse, or a high incidence of new *P. falciparum *infections. Furthermore, since the proportion of reinfections and relapses observed in clinical trials is dependent upon the efficacy of the drug and its pharmacokinetic properties, the potential bias introduced by methodologies has implications for the comparative analysis of antimalarials.

Given the variations in study methods, survival analysis remains the preferred approach for monitoring *in vivo *efficacy. First, survival analysis allows for all available data to contribute to the analysis, thus increasing the precision of the derived estimates. Second, it avoids systematic biases introduced by dropping from the analysis patients who do not complete follow-up (PP) or classifying failures as patients who do not complete follow-up (ITT). Finally, survival analysis allows for data from patients with different follow-up periods to be combined to generate efficacy estimates at different time points, thus enabling direct comparison between studies with different lengths of follow-up [7].

Over the last decade it has become evident that the wider availability of highly effective anti-malarial regimens must be an integral part of any realistic approach to achieving the global elimination of malaria [13]. Current international guidelines advocate that new anti-malarial treatments should be introduced only if they yield cure rates greater than 90%. Once introduced, the efficacy of such novel regimens needs to be monitored regularly in order to detect early signs of declining efficacy. Even small fluctuations in risk estimates or wide confidence intervals can have huge implications for policy makers. In order to monitor temporal and spatial trends in anti-malarial efficacy, *in vivo *efficacy data need to be collated at an individual patient level and standardized analytical tools applied in a transparent and systematic manner [7]. The recently launched WorldWide Antimalarial Resistance Network (WWARN – ), aims to do precisely that; gather global anti-malarial efficacy data and provide open access to their uniform interpretation.

## Competing interests

The authors declare that they have no competing interests.

## Authors' contributions

GD, FN and RNP supervised the clinical studies. WV, GD and RNP analysed the data. All authors contributed to the drafting of the manuscript.

## Supplementary Material

Additional file 1 **Characteristics and treatment outcomes of the clinical trials**. Abbreviations: AL = artemether-lumefantrine; AM = artemether; AP: atovaquone-proguanil; AQ = amodiaquine; AS = artesunate; CQ = chlorquine; DP = dihydroartemisinin-piperaquine; MQ = mefloquine; SP = sulfadoxine-pyramethamine; ACPR = adequate clinical and parasitological response; ETF = early treatment failure; LCF = late clinical failure; LPF = late parasitological failure Click here for file

Additional file 2 **Estimates of the risk of failure according to treatment arm, derived by intention to treat (ITT), modified Intention to Treat (mITT) and per protocol (PP) analysis methods**. Abbreviations: AL = artemether-lumefantrine; AM = artemether; AP: atovaquone-proguanil; AQ = amodiaquine; AS = artesunate; CQ = chlorquine; DP = dihydroartemisinin-piperaquine; MQ = mefloquine; SP = sulfadoxine-pyramethamine Click here for file

## References

[B1] Collins WJ, Greenhouse B, Rosenthal PJ, Dorsey G (2006). The use of genotyping in antimalarial clinical trials: a systematic review of published studies from 1995–2005. Malar J.

[B2] WHO Guidelines for the treatment of malaria. Document No WHO/HTM/MAL/20061108.

[B3] Guthmann JP, Pinoges L, Checchi F, Cousens S, Balkan S, van Herp M, Legros D, Olliaro P (2006). Methodological issues in the assessment of antimalarial drug treatment: analysis of 13 studies in eight African countries from 2001 to 2004. Antimicrob Agents Chemother.

[B4] Borrmann S, Peto T, Snow RW, Gutteridge W, White NJ (2008). Revisiting the dsign of phase III clinical trials of antimalarial drugs for uncomplicated *Plasmodium falciparum *malaria. PLoS Med.

[B5] Ashley EA, Pinoges L, Turyakira E, Dorsey G, Checchi F, Bukirwa H, Broek I van den, Zongo I, Urruta PP, van Herp M, Balkan S, Taylor WR, Olliaro P, Guthmann JP (2008). Different methodological approaches to the assessment of in vivo efficacy of three artemisinin-based combination antimalarial treatments for the treatment of uncomplicated falciparum malaria in African children. Malar J.

[B6] (2007). Methods and techniques for clinical trials on antimalarial drug efficacy: genotyping to identify parasite populations. Informal consultation organized by the Medicines for Malaria Venture and cosponsored by the World Health Organization.

[B7] Price RN, Dorsey G, Ashley EA, Barnes KI, Baird JK, d'Alessandro U, Guerin PJ, Laufer MK, Naidoo I, Nosten F, Olliaro P, Plowe CV, Ringwald P, Sibley CH, Stepniewska K, White NJ (2007). World Antimalarial Resistance Network I: clinical efficacy of antimalarial drugs. Malar J.

[B8] WHO Assessment and monitoring of antimalarial drug efficacy for the treatment of uncomplicated falciparum malaria. Document No WHO/HTM/RBM200350.

[B9] Nosten F, McGready R, Simpson JA, Thwai KL, Balkan S, Cho T, Hkirijaroen L, Looareesuwan S, White NJ (1999). Effects of *Plasmodium vivax *malaria in pregnancy. Lancet.

[B10] Luxemburger C, Thwai KL, White NJ, Webster HK, Kyle DE, Maelankirri L, Chongsuphajaisiddhi T, Nosten F (1996). The epidemiology of malaria in a Karen population on the western border of Thailand. Trans R Soc Trop Med Hyg.

[B11] Brockman A, Paul RE, Anderson TJ, Hackford I, Phaiphun L, Looareesuwan S, Nosten F, Day KP (1999). Application of genetic markers to the identification of recrudescent *Plasmodium falciparum *infections on the northwestern border of Thailand. Am J Trop Med Hyg.

[B12] Stepniewska K, White NJ (2006). Some considerations in the design and interpretation of antimalarial drug trials in uncomplicated falciparum malaria. Malar J.

[B13] Greenwood BM (2008). Control to elimination: implications for malaria research. Trends Parasitol.

[B14] Zongo I, Dorsey G, Rouamba N, Dokomajilar C, Lankoande M, Ouedraogo JB, Rosenthal PJ (2005). Amodiaquine, sulfadoxine-pyrimethamine, and combination therapy for uncomplicated falciparum malaria: a randomized controlled trial from Burkina Faso. Am J Trop Med Hyg.

[B15] Zongo I, Dorsey G, Rouamba N, Tinto H, Dokomajilar C, Guiguemde RT, Rosenthal PJ, Ouedraogo JB (2007). Artemether-lumefantrine versus amodiaquine plus sulfadoxine-pyrimethamine for uncomplicated falciparum malaria in Burkina Faso: a randomised non-inferiority trial. Lancet.

[B16] Yeka A, Banek K, Bakyaita N, Staedke SG, Kamya MR, Talisuna A, Kironde F, Nsobya SL, Kilian A, Slater M, Reingold A, Rosenthal PJ, Wabwire-Mangen F, Dorsey G (2005). Artemisinin versus nonartemisinin combination therapy for uncomplicated malaria: randomized clinical trials from four sites in Uganda. PLoS Medicine.

[B17] Staedke SG, Mpimbaza A, Kamya MR, Nzarubara BK, Dorsey G, Rosenthal PJ (2004). Combination treatments for uncomplicated falciparum malaria in Kampala, Uganda: randomised clinical trial. Lancet.

[B18] Dorsey G, Staedke S, Clark TD, Njama-Meya D, Nzarubara B, Maiteki-Sebuguzi C, Dokomajilar C, Kamya MR, Rosenthal PJ (2007). Combination therapy for uncomplicated falciparum malaria in Ugandan children: a randomized trial. JAMA.

[B19] Bakyaita N, Dorsey G, Yeka A, Banek K, Staedke SG, Kamya MR, Talisuna A, Kironde F, Nsobya S, Kilian A, Reingold A, Rosenthal PJ, Wabwire-Mangen F (2005). Sulfadoxine-pyrimethamine plus chloroquine or amodiaquine for uncomplicated falciparum malaria: a randomized, multisite trial to guide national policy in Uganda. Am J Trop Med Hyg.

[B20] Bukirwa H, Yeka A, Kamya MR, Talisuna A, Banek K, Bakyaita N, Rwakimari JB, Rosenthal PJ, Wabwire-Mangen F, Dorsey G, Staedke SG (2006). Artemisinin combination therapies for treatment of uncomplicated malaria in Uganda. PLoS Clin Trials.

[B21] Zongo I, Dorsey G, Rouamba N, Dokomajilar C, Sere Y, Rosenthal PJ, Ouedraogo JB (2007). Randomized comparison of amodiaquine plus sulfadoxine-pyrimethamine, artemether-lumefantrine, and dihydroartemisinin-piperaquine for the treatment of uncomplicated Plasmodium falciparum malaria in Burkina Faso. Clin Infect Dis.

[B22] Kamya MR, Yeka A, Bukirwa H, Lugemwa M, Rwakimari JB, Staedke SG, Talisuna AO, Greenhouse B, Nosten F, Rosenthal PJ, Wabwire-Mangen F, Dorsey G (2007). Artemether-lumefantrine versus dihydroartemisinin-piperaquine for treatment of malaria: a randomized trial. PLoS Clin Trials.

[B23] Yeka A, Dorsey G, Kamya MR, Talisuna A, Lugemwa M, Rwakimari JB, Staedke SG, Rosenthal PJ, Wabwire-Mangen F, Bukirwa H (2008). Artemether-lumefantrine versus dihydroartemisinin-piperaquine for treating uncomplicated malaria: a randomized trial to guide policy in Uganda. PLoS ONE.

[B24] van Vugt M, Ezzet F, Nosten F, Gathmann I, Wilairatana P, Looareesuwan S, White NJ (1999). No evidence of cardiotoxicity during antimalarial treatment with artemether-lumefantrine. Am J Trop Med Hyg.

[B25] van Vugt M, Leonardi E, Phaipun L, Slight T, Thway KL, McGready R, Brockman A, Villegas L, Looareesuwan S, White NJ, Nosten F (2002). Treatment of uncomplicated multidrug-resistant falciparum malaria with artesunate-atovaquone-proguanil. Clin Infect Dis.

[B26] Price RN, Uhlemann AC, van Vugt M, Brockman A, Hutagalung R, Nair S, Nash D, Singhasivanon P, Anderson TJ, Krishna S, White NJ, Nosten F (2006). Molecular and pharmacological determinants of the therapeutic response to artemether-lumefantrine in multidrug-resistant Plasmodium falciparum malaria. Clin Infect Dis.

[B27] van Vugt M, Looareesuwan S, Wilairatana P, McGready R, Villegas L, Gathmann I, Mull R, Brockman A, White NJ, Nosten F (2000). Artemether-lumefantrine for the treatment of multidrug-resistant falciparum malaria. Trans R Soc Trop Med Hyg.

[B28] Carrara VI, Barends M, Ashley EA, Price RN, Brockman A, Anderson TJ, Stepniewska K, McGready R, Phaiphun L, Proux S, Preechapkornkul P, Zwang J, Imwong M, Pukritayakamee S, Singhasivanon S, White NJ, Nosten F (2009). The in vivo efficacy and in vitro sensitivity of artesunate and mefloquine during 12 years of continuous deployment on the Thai-Myanmar border. PLoS ONE.

[B29] Price RN, Nosten F, Luxemburger C, van Vugt M, Phaipun L, Chongsuphajaisiddhi T, White NJ (1997). Artesunate/mefloquine treatment of multi-drug resistant falciparum malaria. Trans R Soc Trop Med Hyg.

[B30] Ashley EA, Krudsood S, Phaiphun L, Srivilairit S, McGready R, Leowattana W, Hutagalung R, Wilairatana P, Brockman A, Looareesuwan S, Nosten F, White NJ (2004). Randomized, controlled dose-optimization studies of dihydroartemisinin-piperaquine for the treatment of uncomplicated multidrug-resistant falciparum malaria in Thailand. J Infect Dis.

[B31] van Vugt M, Brockman A, Gemperli B, Luxemburger C, Gathmann I, Royce C, Slight T, Looareesuwan S, White NJ, Nosten F (1998). Randomized comparison of artemether-benflumetol and artesunate-mefloquine in treatment of multidrug-resistant falciparum malaria. Antimicrob Agents Chemother.

[B32] Ashley EA, Lwin KM, McGready R, Simon WH, Phaiphun L, Proux S, Wangseang N, Taylor W, Stepniewska K, Nawamaneerat W, Thwai KL, Barends M, Leowattana W, Olliaro P, Singhasivanon P, White NJ, Nosten F (2006). An open label randomized comparison of mefloquine-artesunate as separate tablets vs. a new co-formulated combination for the treatment of uncomplicated multidrug-resistant falciparum malaria in Thailand. Trop Med Int Health.

[B33] Price RN, Nosten F, Luxemburger C, Kham A, Brockman A, Chongsuphajaisiddhi T, White NJ (1995). Artesunate versus artemether in combination with mefloquine for the treatment of multidrug-resistant falciparum malaria. Trans R Soc Trop Med Hyg.

[B34] Nosten F, Luxemburger C, ter Kuile FO, Woodrow C, Eh JP, Chongsuphajaisiddhi T, White NJ (1994). Treatment of multidrug-resistant Plasmodium falciparum malaria with 3-day artesunate-mefloquine combination. J Infect Dis.

[B35] Luxemburger C, Nosten F, Raimond SD, Chongsuphajaisiddhi T, White NJ (1995). Oral artesunate in the treatment of uncomplicated hyperparasitemic falciparum malaria. Am J Trop Med Hyg.

