# Supplementary material for: The effect of varying analytical methods on estimates of anti-malarial clinical efficacy
Source: Malar J. 2009 Apr 22;8:77. doi: 10.1186/1475-2875-8-77 (PMC2679050; doi:10.1186/1475-2875-8-77)
Supplement: Additional file 1 — Characteristics and treatment outcomes of the clinical trials. Abbreviations: AL = artemether-lumefantrine; AM = artemether; AP: atovaquone-proguanil; AQ = amodiaquine; AS = artesunate; CQ = chlorquine; DP = dihydroartemisinin-piperaquine; MQ = mefloquine; SP = sulfadoxine-pyramethamine; ACPR = adequate clinical and parasitological response; ETF = early treatment failure; LCF = late clinical failure; LPF = late parasitological failure [file 1475-2875-8-77-S1.doc]

| **Location** | **Duration** | **Transmission Intensity** | **Study Drugs** | **Total Enrolled** | **Follow-up Interrupted** | **ACPR** | **ETF** | **LCF/LPF** | | | | | |
| --- | --- | --- | --- | --- | --- | --- | --- | --- | --- | --- | --- | --- | --- |
|  |  |  |  |  |  |  |  | **Recrudescence** | | ***P. falciparum***  **New Infection** | | **Genotyping Unsuccessful** | **Non-falciparum New infection** |
| Burkina Faso [14] | 28 | Moderate | AQ, AQ+SP, SP | 944 | 113 | 735 | 9 | 34 | | 40 | | 13 | 0 |
| Burkina Faso [15] | 28 | Moderate | AL, AQ+SP | 521 | 43 | 430 | 1 | 4 | | 43 | | 0 | 0 |
| Apac, Uganda [16] | 28 | High | AQ+AS, AQ+SP, CQ+SP | 542 | 10 | 252 | 0 | 61 | | 206 | | 13 | 0 |
| Arua, Uganda [16] | 28 | High | AQ+AS, AQ+SP, CQ+SP | 534 | 10 | 188 | 17 | 75 | | 232 | | 12 | 0 |
| Jinja, Uganda [16] | 28 | Moderate | AQ+AS, AQ+SP, CQ+SP | 543 | 27 | 328 | 6 | 77 | | 95 | | 10 | 0 |
| Kampala, Uganda [17] | 28 | Moderate | AQ+AS, AQ+SP, CQ+SP | 400 | 11 | 266 | 5 | 56 | | 52 | | 10 | 0 |
| Kampala, Uganda [18] | 28 | Moderate | AL, AQ+AS, AQ+SP | 687 | 24 | 544 | 11 | 35 | | 69 | | 1 | 3 |
| Kanungo, Uganda [19] | 28 | Moderate | AQ+SP, CQ+SP | 367 | 10 | 108 | 18 | 129 | | 100 | | 2 | 0 |
| Kyenjojo, Uganda [19] | 28 | Moderate | AQ+SP, CQ+SP | 365 | 14 | 147 | 14 | 61 | | 115 | | 14 | 0 |
| Mubende, Uganda [19] | 28 | Moderate | AQ+SP, CQ+SP | 373 | 19 | 135 | 11 | 64 | | 133 | | 11 | 0 |
| Tororo, Uganda [16] | 28 | High | AQ+AS, AQ+SP, CQ+SP | 541 | 22 | 135 | 8 | 77 | | 290 | | 9 | 0 |
| Tororo, Uganda [20] | 28 | High | AL, AQ+AS | 408 | 5 | 165 | 1 | 16 | | 217 | | 4 | 0 |
| Burkina Faso [21] | 42 | Moderate | AL, AQ+SP, DP | 559 | 42 | 429 | 9 | 9 | | 70 | | 0 | 0 |
| Apac, Uganda [22] | 42 | High | AL, DP | 421 | 4 | 206 | 0 | 41 | | 156 | | 1 | 13 |
| Kanungo, Uganda [23] | 42 | Moderate | AL, DP | 414 | 6 | 317 | 1 | 13 | | 46 | | 7 | 24 |
| Mae Sod, Thailand [24] | 28 | Low | AL | 358 | 46 | 261 | 0 | 22 | | 3 | | 1 | 25 |
| Mae Sod, Thailand [25] | 42 | Low | AP+AS, AP, MQ+AS | 1586 | 173 | 1118 | 0 | 20 | | 42 | | 11 | 222 |
| Mae Sod, [26] | 42 | Low | AL | 592 | 68 | 277 | 0 | 14 | | 40 | | 24 | 169 |
| Mae Sod, Thailand [27] | 42 | Low | AL, MQ+AS | 170 | 42 | 97 | 0 | 3 | | 1 | | 0 | 27 |
| Mae Sod, Thailand [26, 28] | 42 | Low | AL, MQ+AS | 795 | 48 | 481 | 0 | 23 | | 51 | | 8 | 184 |
| Mae Sod, Thailand [29] | 42 | Low | MQ+AS | 1019 | 227 | 651 | 0 | 116 (genotyping not done) | | | | | 25 |
| Mae Sod, Thailand [30] | 63 | Low | DP, MQ+AS | 1026 | 102 | 663 | 2 | 20 | 86 | | 9 | | 144 |
| Mae Sod, Thailand [31] | 63 | Low | AL, MQ+AS | 606 | 82 | 308 | 0 | 41 | 22 | | 17 | | 136 |
| Mae Sod, Thailand [32] | 63 | Low | MQ+AS | 493 | 67 | 228 | 0 | 33 | 61 | | 13 | | 91 |
| Mae Sod, Thailand [28] | 63 | Low | MQ+AS | 187 | 45 | 74 | 0 | 10 | 17 | | 16 | | 25 |
| Mae Sod, Thailand [10] | 63 | Low | MQ+AS | 34 | 1 | 22 | 0 | 9 (genotyping not done) | | | | | 2 |
| Mae Sod, Thailand [33] | 63 | Low | MQ, MQ+AM, MQ+AS | 548 | 137 | 239 | 5 | 127 (genotyping not done) | | | | | 40 |
| Mae Sod, Thailand [34] | 63 | Low | MQ, MQ+AS | 346 | 73 | 172 | 6 | 67 (genotyping not done) | | | | | 28 |
| Mae Sod, Thailand [35] | 63 | Low | MQ+AS | 30 | 6 | 13 | 0 | 9 (genotyping not done) | | | | | 2 |
