# Supplementary material for: The effect of varying analytical methods on estimates of anti-malarial clinical efficacy
Source: Malar J. 2009 Apr 22;8:77. doi: 10.1186/1475-2875-8-77 (PMC2679050; doi:10.1186/1475-2875-8-77)
Supplement: Additional file 2 — Estimates of the risk of failure according to treatment arm, derived by intention to treat (ITT), modified Intention to Treat (mITT) and per protocol (PP) analysis methods. Abbreviations: AL = artemether-lumefantrine; AM = artemether; AP: atovaquone-proguanil; AQ = amodiaquine; AS = artesunate; CQ = chlorquine; DP = dihydroartemisinin-piperaquine; MQ = mefloquine; SP = sulfadoxine-pyramethamine [file 1475-2875-8-77-S2.doc]

|  |  |  |  | **Risk of Failure Estimates % [CI 95%]** | | | | | | | |
| --- | --- | --- | --- | --- | --- | --- | --- | --- | --- | --- | --- |
|  |  | **Study** |  | **Unadjusted** | | | **Adjusted** | | | | |
| **Location** | **Duration** | **Drugs** | **N** | **ITT** | **mITT** | **PP** | **ITT** | | **mITT** | | **PP** |
| Burkina Faso [14] | 28 | AQ | 317 | 28.4  [23.4-33.4] | 19.0  [14.9-24.1] | 19.2  [14.6-23.9] | 20.9  [16.3-25.3] | | 9.0  [6.1-13.0] | | 9.9  [6.2-13.6] |
|  |  | AQ+SP | 323 | 16.1  [12.1-20.1] | 4.8  [2.9-8.0] | 4.9  [2.4-7.4] | 14.2  [10.4-18.1] | | 2.1  [0.9-4.5] | | 2.2  [0.4-3.9] |
|  |  | SP | 304 | 22.0  [17.4-26.7] | 10.3  [7.2-14.6] | 10.6  [6.8-14.3] | 18.8  [14.3-23.2] | | 4.4  [2.5-7.6] | | 4.8  [2.1-7.5] |
| Burkina Faso [15] | 28 | AL | 261 | 20.3  [15.4-25.2] | 15.1  [11.2-20.2] | 15.1  [10.6-19.6] | 7.7  [4.4-10.9] | | 1.7  [0.6-4.5] | | 1.9  [0.0-3.7] |
|  |  | AQ+SP | 260 | 14.6  [10.3-18.9] | 4.7  [2.6-8.3] | 4.7  [2.0-7.5] | 10.8  [7.0-14.6] | | 0.4  [0.1-2.7] | | 0.4  [-0.4-1.3] |
| Apac, Uganda [16] | 28 | AQ+AS | 174 | 54.0  [46.5-61.5] | 53.5  [46.2-61.1] | 53.5  [46.0-61.0] | 12.1  [7.2-17.0] | | 10.4  [6.4-16.8] | | 15.8  [8.3-23.3] |
|  |  | AQ+SP | 183 | 38.8  [31.7-45.9] | 37.1  [30.5-44.6] | 37.1  [29.9-44.2] | 11.5  [6.8-16.1] | | 7.3  [4.2-12.5] | | 9.7  [4.4-15.0] |
|  |  | CQ+SP | 185 | 67.6  [60.8-74.4] | 67.0  [60.1-73.7] | 67.0  [60.1-73.9] | 22.7  [16.6-28.8] | | 24.8  [18.2-33.2] | | 36.2  [26.3-46.1] |
| Arua, Uganda [16] | 28 | AQ+AS | 174 | 51.7  [44.2-59.2] | 50.7  [43.4-58.3] | 50.9  [43.3-58.4] | 11.5  [6.7-16.3] | | 7.8  [4.5-13.4] | | 12.5  [5.8-19.2] |
|  |  | AQ+SP | 180 | 55.0  [47.7-62.3] | 53.4  [46.3-61.0] | 53.4  [46.0-60.9] | 15.6  [10.2-20.9] | | 13.5  [8.7-20.5] | | 19.0  [11.2-26.8] |
|  |  | CQ+SP | 180 | 87.2  [82.3-92.1] | 86.9  [81.5-91.3] | 87.2  [82.2-92.1] | 36.7  [29.6-43.8] | | 50.4  [40.2-61.6] | | 72.6  [62.9-82.4] |
| Jinja, Uganda [16] | 28 | AQ+AS | 189 | 22.8  [16.7-28.8] | 19.2  [14.2-25.7] | 19.3  [13.5-25.1] | 9.5  [5.3-13.7] | | 4.1  [2.0-8.5] | | 4.6  [1.2-7.9] |
|  |  | AQ+SP | 186 | 33.3  [26.5-40.2] | 28.6  [22.5-35.9] | 28.7  [21.9-35.5] | 19.4  [13.6-25.1] | | 12.8  [8.6-19.1] | | 14.5  [8.7-20.3] |
|  |  | CQ+SP | 168 | 65.5  [58.2-72.7] | 63.3  [55.9-70.6] | 64.0  [56.5-71.5] | 39.3  [31.8-46.7] | | 38.5  [30.9-47.3] | | 48.7  [39.3-58.0] |
| Kampala, Uganda [17] | 28 | AQ+AS | 134 | 19.4  [12.6-26.2] | 17.6  [12.0-25.2] | 17.6  [11.0-24.2] | 7.5  [3.0-12.0] | | 3.3  [1.2-8.4] | | 3.6  [0.1-7.1] |
|  |  | AQ+SP | 134 | 20.9  [13.9-27.9] | 17.8  [12.2-25.5] | 17.8  [11.1-24.5] | 13.4  [7.6-19.3] | | 9.4  [5.4-15.9] | | 10.2  [4.6-15.7] |
|  |  | CQ+SP | 132 | 60.6  [52.2-69.1] | 59.6  [51.3-68.1] | 59.7  [51.1-68.3] | 40.9  [32.4-49.4] | | 39.6  [31.1-49.4] | | 46.4  [36.3-56.5] |
| Kampala, Uganda [18] | 28 | AL | 202 | 9.4  [5.3-13.5] | 6.7  [3.9-11.2] | 6.7  [3.2-10.3] | 4.0  [1.2-6.7] | | 1.0  [0.3-4.0] | | 0.1  [-0.4-2.6] |
|  |  | AQ+AS | 232 | 20.7  [15.4-25.9] | 17.4  [13.1-23.1] | 17.5  [12.5-22.5] | 8.6  [5.0-12.3] | | 4.5  [2.5-8.3] | | 5.2  [2.0-8.3] |
|  |  | AQ+SP | 253 | 28.9  [23.2-34.5] | 26.1  [21.1-32.1] | 26.2  [20.7-31.8] | 17.0  [12.3-21.7] | | 14.1  [10.3-19.2] | | 15.9  [11.0-20.8] |
| Kanungo, Uganda [19] | 28 | AQ+SP | 190 | 56.8  [49.7-63.9] | 55.2  [48.1-62.4] | 55.4  [48.2-62.7] | 33.2  [26.4-39.9] | | 31.5  [25.1-39.1] | | 40.1  [31.8-48.5] |
|  |  | CQ+SP | 177 | 85.3  [80.0-90.6] | 84.8  [79.1-89.7] | 85.0  [79.6-90.3] | 54.2  [46.8-61.6] | | 64.0  [55.4-72.5] | | 78.0  [70.4-85.6] |
| Kyenjojo, Uganda [19] | 28 | AQ+SP | 190 | 45.8  [38.6-52.9] | 42.8  [36.0-50.4] | 43.1  [35.8-50.4] | 23.2  [17.1-29.2] | | 15.1  [10.4-21.6] | | 19.5  [12.6-26.5] |
|  |  | CQ+SP | 175 | 74.9  [68.4-81.3] | 74.0  [67.2-80.3] | 74.1  [67.5-80.8] | 33.7  [26.6-40.8] | | 38.3  [30.1-47.8] | | 53.2  [42.9-63.5] |
| Mubende, Uganda [19] | 28 | AQ+SP | 180 | 49.4  [42.1-56.8] | 47.0  [40.0-54.7] | 47.4  [39.9-54.9] | 16.1  [10.7-21.5] | | 12.4  [8.0-18.8] | | 17.3  [10.1-24.4] |
|  |  | CQ+SP | 193 | 77.2  [71.2-83.2] | 75.5  [69.1-81.5] | 75.7  [69.4-82.0] | 39.4  [32.4-46.3] | | 35.2  [28.0-43.5] | | 56.0  [46.1-65.9] |
| Tororo, Uganda [16] | 28 | AQ+AS | 194 | 76.3  [70.3-82.2] | 74.5  [68.0-80.6] | 74.7  [68.4-81.1] | 16.0  [10.8-21.2] | | 12.8  [7.6-20.9] | | 24.6  [0.13-0.36] |
|  |  | AQ+SP | 181 | 61.9  [54.7-69.0] | 60.5  [53.4-67.8] | 60.6  [53.3-67.9] | 21.0  [15.0-27.0] | | 18.5  [13.0-26.0] | | 28.1  [19.0-37.3] |
|  |  | CQ+SP | 166 | 88.0  [82.9-93.0] | 87.6  [81.9-92.1] | 87.7  [82.5-92.8] | 28.3  [21.4-35.2] | | 37.4  [28.1-48.6] | | 68.3  [56.4-80.1] |
| Tororo, Uganda [20] | 28 | AL | 204 | 52.5  [45.5-59.4] | 52.0  [45.3-59.0] | 52.0  [45.0-58.9] | 7.8  [4.1-11.6] | | 7.0  [3.9-12.3] | | 10.2  [4.4-16.0] |
|  |  | AQ+AS | 204 | 66.7  [60.1-73.2] | 66.0  [59.5-72.5] | 66.2  [59.6-72.8] | 4.9  [1.9-7.9] | | 4.0  [1.8-8.9] | | 8.1  [1.7-14.5] |
| Burkina Faso [21] | 42 | AL | 188 | 35.6  [28.7-42.5] | 30.9  [24.7-38.3] | 31.3  [24.3-38.2] | 10.1  [5.8-14.5] | | 4.1  [2.0-8.5] | | 5.5  [1.5-9.5] |
|  |  | AQ+SP | 184 | 19.0  [13.3-24.7] | 11.6  [7.6-17.4] | 11.8  [6.9-16.8] | 12.0  [7.2-16.7] | | 3.9  [1.9-8.0] | | 4.5  [1.2-7.8] |
|  |  | DP | 187 | 15.0  [9.8-20.1] | 7.5  [4.4-12.5] | 7.6  [3.6-11.5] | 10.2  [5.8-14.5] | | 2.2  [0.8-5.8] | | 2.5  [0.1-4.9] |
| Apac, Uganda [22] | 42 | AL | 210 | 52.4  [45.6-59.2] | 52.7  [46.1-59.8] | 54.8  [47.8-61.8] | 14.8  [9.9-19.6] | | 16.4  [11.5-23.0] | | 23.9  [16.1-31.8] |
|  |  | DP | 211 | 43.6  [36.9-50.3] | 43.2  [36.850.2] | 43.5  [36.7-50.3] | 7.1  [3.6-10.6] | | 6.9  [4.1-11.6] | | 10  [4.8-15.2] |
| Kanungo, Uganda [23] | 42 | AL | 199 | 24.6  [18.6-30.7] | 24.2  [18.7-30.9] | 26.0  [19.5-32.5] | 9.5  [5.4-13.7] | | 5.7  [3.1-10.3] | | 7.1  [2.8-11.4] |
|  |  | DP | 215 | 11.2  [6.9-15.4] | 9.9  [6.6-14.8] | 10.1  [6.0-14.3] | 3.7  [1.2-6.3] | | 2.0  [0.7-5.1] | | 2.1  [0.0-4.2] |
| Mae Sod, Thailand [24] | 28 | AL | 358 | 20.1  [15.9-24.3] | 8.2  [5.6-11.8] | 9.1  [5.7-12.4] | 19.3  [15.2-23.4] | | 6.9  [4.6-10.4] | | 7.8  [4.6-10.9] |
| Mae Sod, Thailand [25] | 42 | AP+AS | 526 | 12.5  [9.7-15.4] | 3.6  [3.8-8.2] | 4.0  [2.0-6.0] | 10.5  [7.8-13.1] | | 0.2  [0.0-1.7] | | 0.3  [-0.3-0.8] |
|  |  | AP | 528 | 13.8  [10.9-16.8] | 5.6  [0.04-0.08] | 6.5  [4.0-9.0] | 11.9  [9.2-14.7] | | 2.4  [1.3-4.3] | | 3.0  [1.2-4.7] |
|  |  | MQ+AS | 532 | 20.1  [16.7-23.5] | 7.1  [5.1-9.9] | 7.6  [5.1-10.1] | 16.2  [13.0-19.3] | | 1.7  [0.8-3.3] | | 2.0  [0.6-3.3] |
| Mae Sod, Thailand [26] | 42 | AL | 592 | 24.7  [21.2-28.1] | 17.7  [14.3-21.6] | 22.0  [17.6-26.3] | 17.9  [14.8-21.0] | | 3.1  [1.8-5.2] | | 4.8  [2.3-7.3] |
| Mae Sod, Thailand [27] | 42 | AL | 127 | 28.3  [20.4-36.3] | 3.6  [1.4-9.4] | 5.8  [0.1-11.5] | 27.6  [19.7-35.4] | | 2.8  [0.9-8.4] | | 4.4  [-0.6-9.4] |
|  |  | MQ+AS | 43 | 23.3  [10.1-36.4] | 0 | 0 | 23.3  [10.1-36.4] | | 0 | | 0 |
| Mae Sod, Thailand [26, 28] | 42 | AL | 310 | 14.5  [10.6-18.5] | 10.5  [7.1-15.3] | 14.6  [9.3-20.0] | 8.7  [5.6-11.9] | | 2.3  [1.0-5.2] | | 3.9  [0.8-7.1] |
|  |  | MQ+AS | 485 | 17.5  [14.1-20.9] | 12.6  [9.9-16.1] | 14.5  [11.0-18.0] | 10.7  [8.0-13.5] | | 3.8  [2.4-6.0] | | 4.8  [2.6-7.1] |
| Mae Sod, Thailand [29] | 42 | MQ+AS | 1019 | 33.7  [30.8-36.6] | 13.0  [11.0-15.4] | 15.1  [12.6-17.7] | genotyping not done | | | | |
| Mae Sod, Thailand [30] | 63 | DP | 686 | 19.7  [16.7-22.7] | 11.3  [9.0-14.1] | 13.0  [10.1-15.9] | 12.0  [9.5-14.4] | 1.1  [0.5-2.3] | | 1.5  [0.4-2.7] | |
|  |  | MA+AS | 343 | 24.6  [20.0-29.1] | 16.0  [12.4-20.6] | 18.9  [14.1-23.6] | 14.9  [11.1-18.7] | 4.7  [2.8-7.6] | | 6.5  [3.3-9.7] | |
| Mae Sod, Thailand [31] | 63 | AL | 302 | 29.1  [24.0-34.3] | 22.5  [17.7-28.4] | 29.5  [22.9-36.0] | 25.2  [20.2-30.1] | 12.9  [9.3-17.8] | | 19.8  [13.7-25.9] | |
|  |  | MA+AS | 305 | 24.3  [19.5-29.2] | 10.2  [7.0-15.0] | 12.1  [7.5-16.7] | 21.1  [16.4-25.7] | 3.3  [1.6-6.5] | | 4.4  [1.4-7.4] | |
| Mae Sod, Thailand [32] | 63 | MQ+AS | 500 | 35.3  [31.1-39.5] | 26.1  [22.0-30.7] | 31.9  [26.9-37.0] | 22.9  [19.2-26.6] | 7.8  [5.6-10.8] | | 12.6  [8.6-16.7] | |
| Mae Sod, Thailand [28] | 63 | MQ+AS | 187 | 47.1  [39.8-54.3] | 29.5  [22.7-37.8] | 36.8  [27.8-45.6] | 38.0  [30.9-45.0] | 6.2  [3.4-11.3] | | 11.9  [4.8-19.0] | |
| Mae Sod, Thailand [10] | 63 | MQ+AS | 34 | 29.4  [13.3-45.5] | 27.3  [15.2-45.9] | 29.0  [12.1-46.0] | genotyping not done | | | | |
| Mae Sod, Thailand [33] | 63 | MQ | 184 | 66.7  [59.8-73.6] | 50.9  [43.0-59.3] | 61.5  [53.1-70.0] | genotyping not done | | | | |
|  |  | MQ+AM | 180 | 48.3  [41.0-55.7] | 18.3  [12.5-26.3] | 22.6  [14.5-30.7] |  |  |  |  |  |
|  |  | MQ+AS | 185 | 32.4  [25.6-39.2] | 19.5  [13.9-27.1] | 20.7  [13.8-27.7] |  |  |  |  |  |
| Mae Sod, Thailand [34] | 63 | MQ | 167 | 54.5  [46.9-62.1] | 45.3  [37.3-54.1] | 51.6  [42.7-60.4] | genotyping not done | | | | |
|  |  | MQ+AS | 179 | 30.7  [23.9-37.6] | 6.5  [3.3-12.8] | 6.7  [2.2-11.3] |  |  |  |  |  |
| Mae Sod, Thailand [35] | 63 | MQ+AS | 30 | 50.0  [31.0-69.0] | 36.6  [20.5-59.7] | 40.9  [18.6-63.2] | genotyping not done | | | | |
